# Supplementary material for: Fluorescence Enhancement in Topologically Optimized Gallium Phosphide All-Dielectric Nanoantennas
Source: Nano Lett. 2024 Feb 14;24(8):2437–43. doi: 10.1021/acs.nanolett.3c03773 (PMC10905999; doi:10.1021/acs.nanolett.3c03773)
Supplement: Supplementary file 1 — nl3c03773_si_001.pdf [file nl3c03773_si_001.pdf]

Supporting Information: Fluorescence enhancement in topologically optimized gallium phosphide all-dielectric nanoantennas

Cynthia Vidal,[*,†] Benjamin Tilmann,[‡] Sunny Tiwari,[¶] T. V. Raziman,[§,†] Stefan A. Maier,[‖,‡,†] Jérôme Wenger,[¶] and Riccardo Sapienza[*,†]

†*The Blackett Laboratory, Department of Physics, Imperial College London, London SW7 2AZ, U.K.*

‡*Nano-Institute Munich, Department of Physics, Ludwig-Maximilians-University Munich, 80539 Munich, Germany*

¶*Aix Marseille Univ, CNRS, Centrale Marseille, Institut Fresnel, 13013 Marseille, France*

§*Department of Mathematics, Imperial College London, London SW7 2AZ, U.K.*

‖*School of Physics and Astronomy, Monash University, Clayton, Victoria 3800, Australia*

E-mail: c.vidal@imperial.ac.uk; r.sapienza@imperial.ac.uk

Methods

Sketch of the nanofabrication procedure

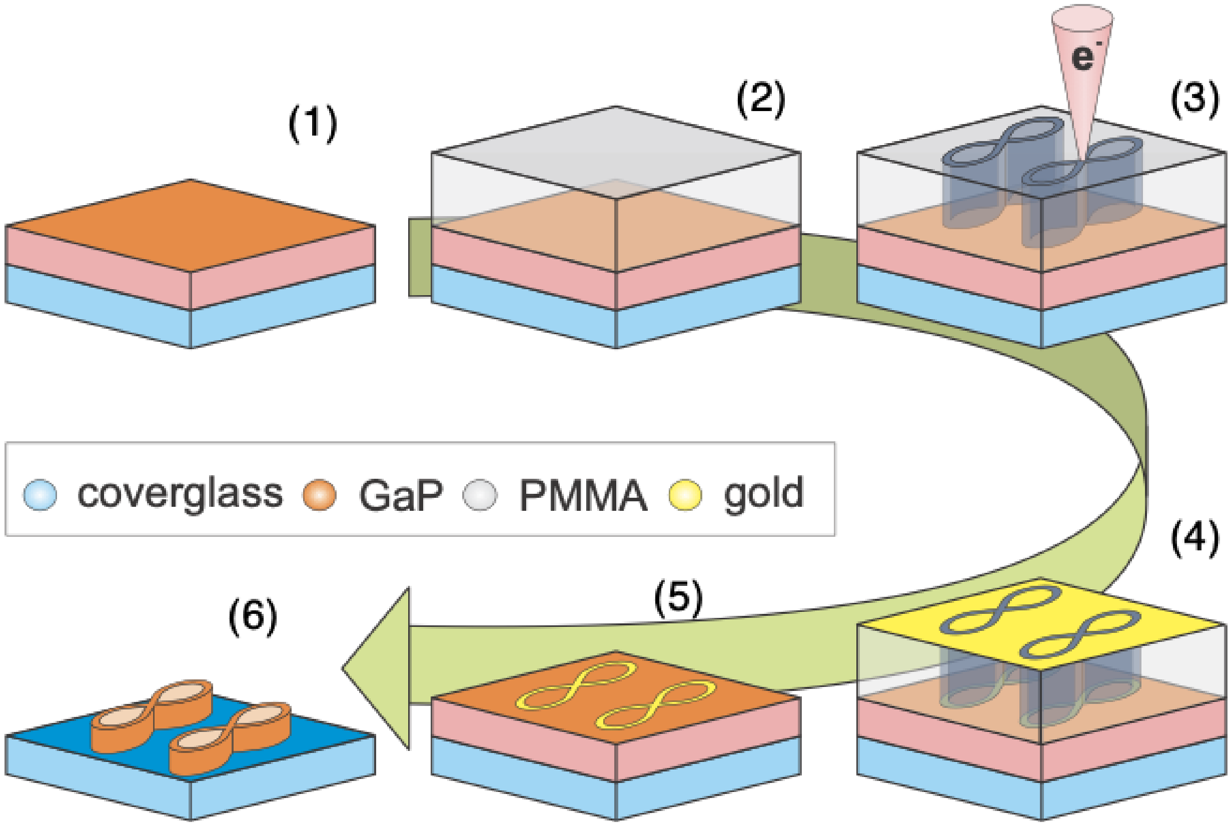

Figure S1: First a 50 nm film of gallium phosphide (GaP) is sputtered onto a glass coverslip (1). A layer of poly(methyl methacrylate) (PMMA) is spin-coated on the GaP film (2) before EBL of the infinity antenna structures is performed (3). The sample is then developed to remove the exposed PMMA and coated with a 40-50 nm thick layer of gold (Au) (4). The unexposed PMMA+Au layer is removed via chemical liftoff thus leaving Au only on the structured areas of GaP (5). Reactive ion etching removes the GaP left unshielded by the Au and a final chemical etching removes the last layer of gold leaving only the structured GaP infinity antenna on the coverslip (6).

Table S1: Dimensions of GaP nanoantennas determined from SEM images. Length and width measured at broadest points of the nanoantennas.

Length (nm)	Width (nm)	Gap (nm)
620	490	45
500	390	35
690	540	25
550	435	25
745	585	30
740	565	25
740	590	35
695	535	15
615	475	35
765	585	35
550	430	30

AFM profile of a gapped nanoantenna

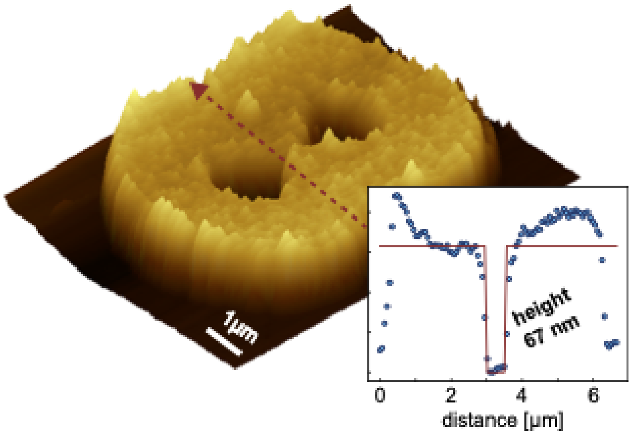

Figure S2: 3D AFM profile of a gapped nanoantenna. Line cut across the center showing a 40 nm gap, 67 nm height

Fluorescence Correlation Spectroscopy: Fit analysis

The temporal correlation of the fluorescence intensity can be written as:

$$G(\tau) = \frac{N^* Q^{*2} G_d^*(\tau) + N_0 Q_0^2 G_{d0}(\tau)}{(N^* Q^* + N_0 Q_0)^2} = \rho_1 G_d^*(\tau) + \rho_2 G_{d0} \tag{1}$$

where N^* is the number of molecules within the gap region with brightness Q^*, and N_0 is the number of molecules with brightness Q_0 diffusing away from the region of interest. ρ_1 and ρ_2 are the amplitude of a 2-species FCS fit while $G_d^*(\tau)$ and $G_{d0}(\tau)$ are the normalized correlation functions for each species taken individually based on a classical three dimensional model:

$$G_{di}(\tau) = \frac{1}{(1 + \tau/\tau_{d,i})\sqrt{1 + s_i^2\, \tau/\tau_{d,i}}} \tag{2}$$

$\tau_{d,i}$ stands for the mean residence time (set by translational diffusion) and s_i is the ratio of transversal to axial dimensions of the analysis volume, whose value is set to $s = 0.2$ and has negligible influence on the estimates of the number of molecules and brightness within the gap (N^*, Q^*).

The number of molecules within the gap N^* and their fluorescence brightness Q^* are extracted from the 2-species FCS fit amplitudes ρ_1 and ρ_2, with the additional knowledge of the total fluorescence intensity $I = N_0 Q_0 + N^* Q^*$ directly measured by our instrument:

$$N^* = \frac{(I - N_0 Q_0)^2}{I^2(\rho_1 + \rho_2) - N_0 Q_0^2} \tag{3}$$

$$Q^* = \frac{I^2(\rho_1 + \rho_2) - N_0 Q_0^2}{(I - N_0 Q_0)} \tag{4}$$

The last step to compute N^* and Q^* is to estimate the number of molecules N_0 and brightness Q_0 for the molecules diffusing away from the nanogap hot spot. To this end, we use the FCS results recorded on the same nanoantenna when the excitation polarization is rotated by 90° to be perpendicular to the dimer axis. As additional control, the fluorescence

brightness Q_0 found with perpendicular polarization is similar to the value found for the confocal reference, while the number of molecule N_0 diffusing away from the hot spot is approximately half that seen in the diffraction-limited confocal volume. We relate this effect to the presence of the glass coverslip interface located at the laser focus which cuts the confocal detection volume by a factor of 2.

Fluorescence Correlation Spectroscopy: Table of fit values

Table S2: List of fitting parameters for FCS fits in Figure 2a,b. I is the fluorescence intensity, ρ_1 and ρ_2 are the values of the ACF at zero delay time considering a model with two diffusion times.

Polarization	Parallel	Perpendicular
I (kcounts/s)	162.9	147.1
$\rho_1 (10^{-3})$	3.8	0.3
$\rho_2 (10^{-3})$	2.0	2.1
N_0	500	
Q_0 (kcounts/s)	0.32	
N^*	0.2	
Volume (zL)	235	
Q^* (kcounts/s)	22.9	
Enhancement	72.4	

Numerical simulations

Model

The geometric structure of the antenna used in the simulation was created by importing the two-dimensional cross-section from a scanning electron micrograph (SEM) and raising it to a height of 50 nm. Antennas with different gap sizes were created by distorting the central region of the SEM to increase the gap size without affecting the other dimensions. We have considered an overall average background refractive index $n_b = 1.41$ to account for both the glass substrate and surrounding aqueous solution. The dielectric function of GaP was taken from Ref. 1.

The brightness enhancement in the nanoantenna results from three processes:[2] (1) excitation enhancement due to the concentration of the electric field, (2) quantum yield enhancement due to the Purcell effect, and (3) increased collection efficiency due to antenna beaming effect. These three processes can be disentangled through numerical simulations and accounted for individually. Here, as the wavelength of interest is sufficiently above the band gap, we neglected the ohmic losses in the antenna.

The enhancement factors of local density of optical states (LDOS), for different combinations of locations of the emitter, were evaluated by performing separate simulations with an electric dipole source for each combination, and obtaining the Purcell factor P.

We computed the total fluorescent enhancement E of the antenna by combining the excitation intensity enhancement F, the LDOS enhancement P, and the collection efficiency modification γ, following the method in Ref. 3

$$E = \frac{\gamma \, P}{I\sigma/k_0 + \left[(P-1)\eta_0 + 1\right]/F} \left(1 + \frac{I\sigma}{k_0}\right) \tag{5}$$

where k_0 is the intrinsic total decay rate of the molecule with intrinsic quantum yield η_0, σ is the absorption cross section, and I is the illumination intensity.For this calculation, the emitter is placed at the centre of the gap, aligned along the gap.

As our measurements were undertaken at a laser fluence well below saturation to guarantee a linear dependence with the laser power, we used the simplification of the low-intensity regime

$$E \approx \frac{\gamma \; P \; F}{1 - \eta_0 + \eta_0 \; P} \, . \tag{6}$$

Contribution of the incident field

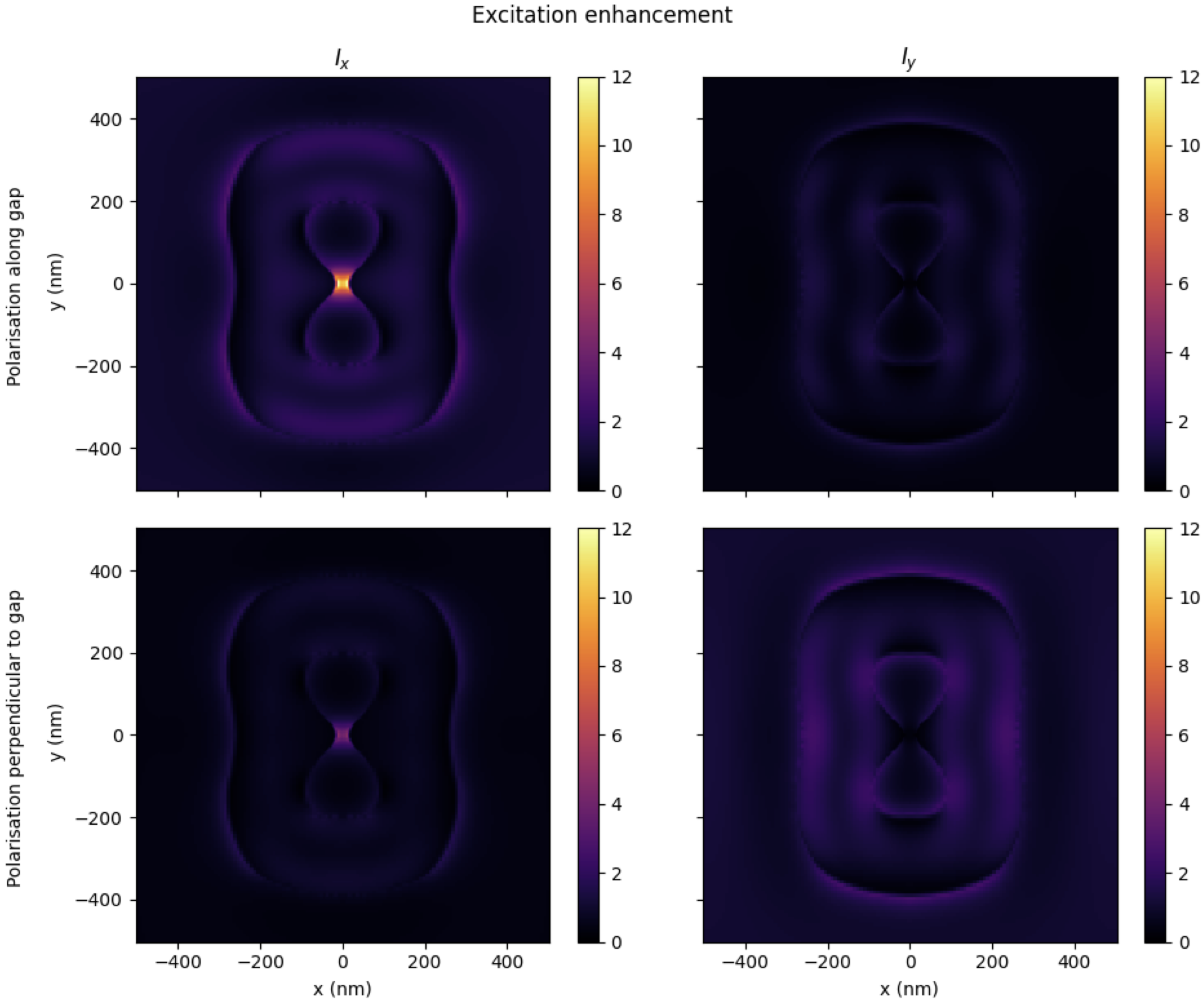

Figure S3: The incident field enhancements, $|E|/|E_0|$, for both incident polarization computed by averaging local enhancements over multiple illuminations within the numerical aperture and treating the polarised intensities according to chapter 3 in Ref. 4

Spectral mode decomposition

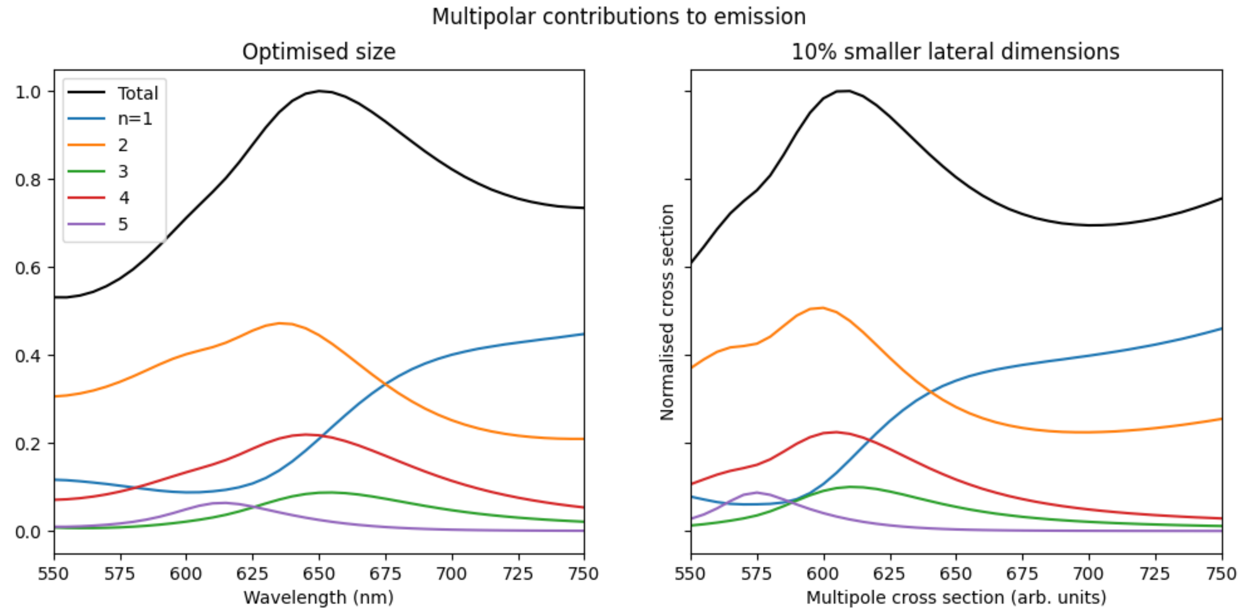

Figure S4: Spectral responses of a nanoantenna with an optimal size (left) and one with a size corresponding to the fabricated ones, i.e. with 10% smaller lateral dimensions (right).

References

(1) Aspnes, D. E.; Studna, A. A. Dielectric functions and optical parameters of Si, Ge, GaP, GaAs, GaSb, InP, InAs, and InSb from 1.5 to 6.0 eV. *Phys. Rev. B* **1983**, *27*, 985–1009.

(2) Regmi, R.; Berthelot, J.; Winkler, P. M.; Mivelle, M.; Proust, J.; Bedu, F.; Ozerov, I.; Begou, T.; Lumeau, J.; Rigneault, H.; García-Parajó, M. F.; Bidault, S.; Wenger, J.; Bonod, N. All-Dielectric Silicon Nanogap Antennas To Enhance the Fluorescence of Single Molecules. *Nano Lett.* **2016**, *16*, 5143–5151.

(3) Kern, A. M.; Meixner, A. J.; Martin, O. J. F. Molecule-Dependent Plasmonic Enhancement of Fluorescence and Raman Scattering near Realistic Nanostructures. *ACS Nano* **2012**, *6*, 9828–9836.

(4) Novotny, L.; Hecht, B. *Principles of Nano-Optics*; Cambridge University Press, 2006.
